# Estimating Infarct Size in the Troponin Era: Why No Biomarker Has Replaced Creatine Kinase-Myocardial Band Area Under the Curve (CK-MB AUC) and Where the Field Must Go Next

**DOI:** 10.7759/cureus.109879

**Published:** 2026-05-29

**Authors:** Alec J Lippmann, Ali Qureshi, Mujtaba Khan, Musa Azhar, Fawaz Qazi, Muhammad Awan, Stuart Tauberg

**Affiliations:** 1 Research, Alabama College of Osteopathic Medicine, Dothan, USA; 2 Research, University of Central Florida, Orlando, USA; 3 Cardiology, Cardiac and Vascular Consultants, The Villages, USA

**Keywords:** area under the curve (auc), biomarkers, cardiac magnetic resonance, ck-mb, creatine kinase-mb, infarct size, myocardial infarction, troponin

## Abstract

Infarct size remains a clinically meaningful determinant of prognosis after myocardial infarction (MI) and an important surrogate endpoint in cardiovascular research. Although cardiac troponins have appropriately replaced creatine kinase-myocardial band (CK-MB) for diagnosis because of superior sensitivity and specificity, they have not replaced the quantitative role historically served by serial CK-MB area-under-the-curve (AUC) analysis. Troponin release is prolonged, assay-dependent, and frequently influenced by baseline myocardial injury, making cumulative troponin exposure difficult to interpret as a direct measure of infarct mass. Imaging modalities, especially late gadolinium enhancement cardiac magnetic resonance (CMR), provide accurate infarct characterization but are limited by cost, access, timing, workflow, and scalability. This narrative review revisits the historical utility of CK-MB AUC, explains why troponin has not provided an equivalent biomarker-based infarct-size estimate, and argues for renewed development of practical biomarker strategies, including kinetic modeling, multi-marker panels, and novel biomarkers designed specifically for infarct-size quantification.

## Introduction and background

Infarct size is a fundamental determinant of outcomes after myocardial infarction (MI), with strong associations with mortality, heart failure, ventricular remodeling, arrhythmia risk, and long-term functional status [1-5]. For this reason, infarct size has long served as a surrogate endpoint in cardiovascular trials and translational studies, allowing investigators to evaluate myocardial salvage and therapeutic efficacy without relying exclusively on hard clinical outcomes. Accurate infarct-size estimation is therefore not merely descriptive; it is central to prognosis, risk stratification, and the evaluation of cardioprotective therapies.

Throughout this review, the area under the curve (AUC) refers to the area under the concentration-time curve generated from serial biomarker measurements. In practice, biomarker values are obtained at prespecified intervals after MI, plotted against time, and integrated, most commonly using the trapezoidal rule, so that the cumulative signal approximates the total amount of biomarker released into the circulation [6,7]. For CK-MB, this cumulative exposure historically served as a practical biochemical surrogate for the burden of myocardial necrosis, whereas a single peak value represents only one point on the release curve.

This article is a narrative review rather than a systematic review. Relevant literature was identified through targeted searches of PubMed/MEDLINE and Google Scholar, review of major guidelines and consensus documents, and backward citation review of key articles. Searches were performed iteratively from database inception through May 2026 using combinations of the following terms: MI, infarct size, creatine kinase-MB, CK-MB, area under the curve, biomarker kinetics, troponin, high-sensitivity troponin, cardiac magnetic resonance, late gadolinium enhancement, SPECT, PET, echocardiography, STEMI, NSTEMI, natriuretic peptides, BNP, NT-proBNP, ventricular remodeling, and prognosis. No formal systematic search strategy, PRISMA flow diagram, prespecified inclusion or exclusion criteria, or risk-of-bias assessment was performed. The conclusions should therefore be interpreted as a narrative synthesis of representative and mechanistically relevant literature rather than as a systematic review or meta-analysis.

Before the troponin era, biomarker-based infarct sizing relied heavily on serial creatine kinase (CK) and CK-myocardial band (MB) measurements. Enzymatic estimates of myocardial necrosis correlated with anatomic, scintigraphic, and angiographic measures of infarct size, and CK-MB release was used in trials and mechanistic studies to quantify myocardial injury over time [8-12]. Although CK-MB was never a perfect diagnostic biomarker, its relatively compact release profile made serial integration useful for estimating the cumulative burden of myocardial necrosis.

Cardiac troponins transformed the diagnosis and classification of MI. Contemporary definitions of MI rely on troponin because it is more sensitive and more myocardial-specific than CK-MB, particularly for non-ST-elevation presentations and small areas of injury [13-16]. This diagnostic success contributed to the clinical decline of CK-MB testing [17]. However, replacement of CK-MB for diagnosis did not solve a separate problem: no troponin-based method has been standardized or broadly validated to replace CK-MB AUC for quantitative infarct-size estimation.

This gap is particularly relevant in non-ST-segment elevation MI (NSTEMI), where infarcts are often smaller, subendocardial, temporally heterogeneous, and clinically less discrete than classic ST-segment elevation MI (STEMI) presentations. Symptom onset may be less precise, angiographic culprit identification may be more ambiguous, and baseline troponin elevation from chronic myocardial injury may complicate interpretation. In a CMR study that included both STEMI and NSTEMI patients, correlations between troponin metrics and infarct mass were substantially weaker in NSTEMI than in STEMI, highlighting that infarct-size estimation in NSTEMI remains an unresolved problem rather than a solved extension of STEMI biomarker logic [18].

The result is an unresolved gap in contemporary practice. CK-MB is less available than it once was, yet troponin has not assumed its historical infarct-sizing role. CK-MB elevation after percutaneous coronary intervention (PCI) and post-revascularization enzyme leak also carried prognostic information, emphasizing that biochemical quantification of myocardial injury remains clinically meaningful beyond diagnosis alone [19,20]. In the absence of a validated biomarker surrogate, infarct-size assessment increasingly depends on imaging, particularly CMR, even though imaging is constrained by cost, infrastructure, timing, and access [21-24]. This review examines why troponin has not replaced CK-MB AUC and where the field should go next.

## Review

Pathobiological rationale for infarct-size quantification

The scientific rationale for measuring infarct size begins with the biology of ischemic cell death. Experimental work demonstrated the wavefront phenomenon of myocardial necrosis, in which irreversible injury progresses from the subendocardium toward the epicardium as the duration of coronary occlusion increases [25]. Infarct size is therefore determined not only by the presence of coronary occlusion but also by ischemic duration, collateral flow, myocardial oxygen demand, area at risk, and the timing and quality of reperfusion.

Reperfusion modifies both tissue injury and biomarker release. Restoration of epicardial flow can accelerate washout of necrosis markers, producing earlier and sometimes higher early biomarker peaks, while incomplete tissue-level reperfusion may be accompanied by no-reflow, microvascular obstruction, hemorrhage, or ongoing injury [26,27]. These pathophysiologic processes explain why infarct-size biomarkers must be interpreted kinetically. A single concentration may reflect sampling time, reperfusion status, and clearance as much as final infarct mass.

A useful infarct-size biomarker should therefore satisfy several conditions. It should be released in proportion to irreversible myocardial necrosis, have a release and clearance profile that permits temporal interpretation, be minimally affected by chronic baseline disease, and be reproducible across clinical laboratories. CK-MB historically approximated these conditions better than many alternatives, particularly when measured serially and interpreted as an integrated concentration-time signal rather than as a single diagnostic threshold.

CK-MB as a surrogate for infarct size

CK-MB remains biologically attractive for infarct-size estimation because its release profile is relatively compact and predictable. CK-MB typically rises within four to six hours after myocardial injury, peaks around 18-24 hours, and returns toward baseline within 48-72 hours [28]. This kinetic pattern approximates the finite interval of acute cardiomyocyte necrosis more closely than biomarkers that remain elevated for many days.

Peak CK-MB has been associated with infarct burden, but a single peak value incompletely captures the dynamic process of biomarker release. Early angiographic and enzymatic studies showed that accumulated CK-MB release correlated with anatomic estimates of infarct size, supporting CK-MB as a quantitative surrogate rather than merely a diagnostic marker [9,29]. Subsequent studies comparing CK-MB with nuclear imaging and CMR similarly demonstrated relationships between CK-MB release, chronic scar size, and wall-motion abnormalities [10-12].

The key historical contribution of CK-MB was serial integration. CK-MB AUC provides a cumulative estimate of myocardial necrosis, reflecting the amount of biomarker released over time rather than a single snapshot of enzyme concentration. Serial CK-MB methods were specifically developed for infarct quantification and validated in frameworks that incorporated imaging, pathology, and kinetic modeling [3,7,8,30]. In a STEMI cohort undergoing primary PCI, Rakowski et al. found that CK-MB at six hours, CK-MB at 12 hours, CK-MB AUC, and maximum CK-MB all correlated strongly with six-month CMR infarct size (r=0.71, r=0.73, r=0.72, and r=0.75, respectively; p<0.001 for all). CK-MB AUC also predicted a large residual infarct with a receiver-operating characteristic AUC of 0.823, using an optimal cutoff of 6241.5 IU/L with 80% sensitivity and 79.5% specificity [6].

Other CMR-based studies provide complementary effect-size data. In the Intracoronary Abciximab and Aspiration Thrombectomy in Patients With Large Anterior Myocardial Infarction (INFUSE-AMI) trial, peak CK-MB correlated with CMR infarct size at 30 days (r=0.67, p<0.001) and inversely with left ventricular ejection fraction (r=-0.56, p<0.001). A peak CK-MB threshold of at least 300 IU/L predicted large infarct size with a receiver operating characteristic (ROC) AUC of 0.88 and left ventricular ejection fraction (LVEF) <=40% with an ROC AUC of 0.78; each additional 100 IU/L increase in CK-MB independently predicted one-year major adverse cardiac events (hazard ratio: 1.42, 95% CI: 1.20-1.67, p<0.001) [31]. In a revascularized non-transmural MI cohort, peak CK-MB correlated strongly with recovery and chronic CMR scar size (r>=0.80 for the overall cohort and r>=0.74 for non-transmural infarcts; p<0.001) and with chronic wall-motion abnormality index (r>=0.75 overall; p<0.001) [12].

CK-MB kinetics are also clinically informative after reperfusion or intervention. Because CK-MB normalizes relatively quickly, recurrent elevation may help identify reinfarction or additional myocardial injury after an initial event. CK-MB kinetics have also been used to assess coronary reperfusion during thrombolytic therapy and after intervention, with early peaking supporting reperfusion and delayed release suggesting persistent occlusion or ongoing injury [10,32]. These characteristics explain why CK-MB retained value for infarct sizing and procedural injury assessment even as troponin became dominant for diagnosis.

The limitations of CK-MB should be acknowledged. CK-MB is less specific than troponin in the presence of skeletal muscle injury and is inferior for detecting small myocardial injury. Nevertheless, for the specific task of infarct-size estimation, CK-MB AUC retains features that troponin has not fully replicated: a shorter release window, a clearer return to baseline, and a longer history of validation against imaging and pathologic reference standards [3,7,33].

Troponin's rise and its failure to replace CK-MB AUC

The introduction of cardiac troponins marked a major advance in the evaluation of suspected MI. Troponin assays provide superior myocardial specificity and sensitivity compared with older biomarkers, and high-sensitivity platforms detect myocardial injury at lower concentrations and earlier time points [13,14]. These properties improved diagnosis and risk stratification, especially in non-ST-elevation acute coronary syndromes [15]. However, diagnostic sensitivity does not automatically translate into quantitative accuracy for infarct-size estimation.

A central limitation of troponin as an infarct-sizing biomarker is its prolonged and complex release profile. Troponin exists in cytosolic and structurally bound pools. Early release may reflect cytosolic leakage, whereas later release reflects ongoing degradation of the myofibrillar apparatus and delayed clearance. After acute MI, troponin may remain elevated for 7-14 days, creating an extended concentration-time tail that extends beyond the discrete window of irreversible necrosis [16,28,34]. By contrast, CK-MB returns toward baseline more rapidly, creating a more interpretable temporal window for AUC analysis.

High-sensitivity troponin assays also detect low-level myocardial injury in many non-ischemic or chronic conditions. Structural heart disease, heart failure, renal dysfunction, supply-demand mismatch, advanced age, and other forms of myocardial injury can produce baseline elevations that complicate serial interpretation [35,36]. When AUC methods are applied to troponin, preexisting or persistent elevations may inflate cumulative values independent of infarct mass.

Troponin release may also show heterogeneous or multiphasic patterns, particularly after reperfusion or PCI. Secondary troponin elevations can reflect reperfusion injury, microvascular obstruction, procedural injury, or delayed cellular breakdown rather than simple infarct expansion [34,37]. These kinetic complexities weaken the assumption that the integrated troponin signal maps cleanly onto the final infarct size.

Assay heterogeneity further limits troponin AUC as a universal quantitative method. Troponin I and troponin T platforms differ in antibody targets, calibration, analytical sensitivity, reporting units, and susceptibility to analytical interference [14,38]. These issues do not prevent troponin from serving as the dominant diagnostic biomarker, but they do complicate attempts to standardize cumulative troponin exposure as an infarct-size endpoint across institutions and studies.

Troponin may still contribute to infarct-size estimation in selected contexts, particularly when its kinetic limitations are controlled. The most plausible settings include STEMI cohorts with a clearly documented symptom onset, early and standardized reperfusion, prespecified serial sampling, preserved or explicitly modeled renal function, absence of substantial chronic baseline troponin elevation, and imaging-anchored validation against CMR or SPECT. In these settings, delayed single-point troponin values, peak values, early slopes, or modeled troponin AUC may contain quantitative information about infarct burden [39-41]. However, these findings have not matured into a universally accepted, assay-independent troponin AUC method for routine infarct-size quantification, especially in heterogeneous NSTEMI populations.

NSTEMI-specific considerations

NSTEMI deserves specific attention because infarct-size estimation is often more challenging than in STEMI. NSTEMI is commonly associated with smaller or subendocardial infarcts, variable timing of coronary occlusion and spontaneous reperfusion, delayed or staged invasive management, and greater overlap with chronic myocardial injury. These features weaken simple assumptions about symptom onset, peak timing, and biomarker washout.

The available CMR literature supports this concern. In a study of 31 STEMI and 30 NSTEMI patients, Giannitsis et al. found that single-point and serial cardiac troponin T values correlated with CMR infarct mass overall, but performance differed substantially by MI subtype. For cTnT on day 4, peak cTnT, and cTnT AUC, correlations with infarct mass were weaker in NSTEMI (r=0.36, r=0.50, and r=0.36, respectively) than in STEMI (r=0.75, r=0.65, and r=0.76, respectively) [18]. This distinction is important: the biomarker strategies most often validated in STEMI cannot simply be assumed to perform equally well in NSTEMI.

Non-transmural infarction studies also reinforce the need for NSTEMI-focused validation. Pöyhönen et al. reported strong associations between peak CK-MB and CMR-defined scar size in revascularized non-transmural MI, including correlations of at least r=0.74 among non-transmural infarcts [12]. These findings suggest that CK-MB and other short-window biomarkers may remain useful in smaller infarcts, but they also underscore the need for contemporary studies specifically designed around NSTEMI populations rather than extrapolated from STEMI cohorts.

Imaging as the contemporary reference standard

CMR with late gadolinium enhancement (LGE) has become the leading imaging approach for infarct-size quantification because it provides high spatial resolution and detailed tissue characterization [42,43]. LGE identifies infarcted myocardium by delayed contrast retention in necrotic or scarred tissue and can distinguish viable from nonviable myocardium with strong histopathologic correlation [21,44]. CMR can also characterize microvascular obstruction, intramyocardial hemorrhage, edema, and other tissue-level consequences of acute MI [45,46].

Nuclear imaging also has a substantial evidence base in infarct-size assessment. Single-photon emission computed tomography (SPECT) imaging with technetium-99m sestamibi has been used extensively in trials, with perfusion-defect size correlating with infarct burden [47]. PET and other multimodality approaches further expand the ability to evaluate ischemia, viability, myocardial blood flow, and post-infarct remodeling [48,49].

Echocardiography remains the most accessible imaging modality and provides bedside assessment of ventricular function, regional wall motion, and complications of MI. Conventional echocardiography is less precise for infarct sizing than CMR, but contrast echocardiography and strain-based approaches can improve detection of regional dysfunction and may be especially useful when CMR is unavailable [50,51].

Despite these strengths, imaging does not eliminate the need for a biomarker surrogate. CMR infarct-size measurement is influenced by timing after MI, edema, inflammation, contrast kinetics, and quantification technique. Cardioprotection trials therefore emphasize standardized CMR timing and methodology, and studies comparing quantification methods show that infarct size and area-at-risk estimates can vary depending on thresholding or contouring approach [52-54]. These factors are manageable in expert core laboratories but difficult to reproduce uniformly in routine care.

Practical barriers are equally important. CMR is expensive, resource-intensive, and dependent on specialized equipment, acquisition protocols, and trained personnel. Cost-effectiveness analyses and access studies emphasize that CMR implementation depends on local infrastructure and health-system resources, and these limitations are especially relevant outside tertiary centers and in low-resource settings [23,24,55,56]. Imaging is indispensable for validation, but it remains imperfect as the sole solution for routine, early, or large-scale infarct-size estimation. Table 1 shows a summary of the major approaches to infarct-size estimation.

**Table 1:** Comparative summary of major approaches to infarct-size estimation BNP: B-type natriuretic peptide; CK-MB AUC: creatine kinase-myocardial band area under the curve; CMR: cardiac magnetic resonance; LGE: late gadolinium enhancement; STEMI: ST-segment elevation myocardial infarction; MI: myocardial infarction

Approach	Timing/signal	Advantages	Limitations	Scalability and validation
CK-MB AUC	Serial concentration-time integration; rise 4-6 h, peak ~18-24 h, return 48-72 h	Compact kinetics; cumulative release reflects necrotic burden; useful for reinfarction and reperfusion assessment	Lower myocardial specificity than troponin; requires serial sampling; less available in many hospitals	Highly scalable when available; strongest historical validation against pathology, nuclear imaging, angiography, and CMR
Troponin	Highly sensitive injury signal; may remain elevated 7-14 d; platform-dependent kinetics	Diagnostic standard for MI; may aid infarct-size estimation in controlled STEMI or imaging-validated cohorts	Baseline elevation, renal dysfunction, chronic injury, prolonged washout, multiphasic release, and assay heterogeneity limit AUC interpretation	Widely available; no standardized or validated assay-independent infarct-size AUC method
CMR with LGE	Tissue characterization during the acute/subacute phase or chronic scar follow-up	Reference standard for infarct size; visualizes edema, microvascular obstruction, hemorrhage, viability, and scar	Cost, access, contraindications, renal/contrast issues, timing effects, and quantification-method variability	Excellent validation; limited scalability for routine early care and large multicenter use without standardization
SPECT/PET	Perfusion, viability, and blood-flow assessment; acute or follow-up imaging, depending on protocol	Extensive SPECT trial experience; PET can quantify myocardial blood flow and balanced ischemia	Radiation exposure; lower spatial resolution for SPECT; PET access and cost limitations	SPECT has strong validation; PET is useful but less universally available
Echocardiography/strain	Bedside functional signal; wall motion and strain can be assessed early and repeatedly	Accessible, inexpensive, repeatable, no radiation; detects ventricular dysfunction and complications	Indirect infarct-size estimate; operator and acoustic-window dependent; less precise than CMR	Highly scalable; best as a complementary functional assessment rather than a definitive infarct-size standard
Natriuretic peptides and emerging biomarkers	BNP/NT-proBNP reflect wall stress and remodeling risk; microRNAs and other markers may capture injury/repair biology	Can complement necrosis markers by adding prognostic and remodeling information	Not direct necrosis markers; confounded by heart failure, renal dysfunction, age, and loading conditions; many candidates remain experimental	Prognostic evidence is growing; infarct-size validation is less mature and requires imaging-anchored studies

Why a biomarker surrogate still matters

A practical biomarker surrogate remains important for both clinical care and research. Clinically, infarct size informs prognosis after MI and helps identify patients at risk for adverse remodeling, heart failure, ventricular arrhythmias, and other complications [4,5,57]. A validated blood-based estimate could support earlier risk stratification when imaging is delayed, unavailable, contraindicated, or impractical.

The strongest argument is not that biomarkers should replace imaging in all circumstances. Rather, biomarkers can complement imaging by providing rapid, repeatable, and scalable assessment. CK-MB measured early after acute MI has been shown to correlate with later CMR-defined infarct size, and simple early infarct-size indices may provide clinically meaningful risk stratification in selected post-STEMI settings [6,58]. Multi-biomarker strategies have also shown promise for early infarct-size estimation [59].

Troponin itself may still contribute to infarct-size estimation if interpreted carefully. Selected studies demonstrate relationships between troponin levels and imaging-defined infarct size, including SPECT-based infarct assessment [40]. However, these associations have not yet matured into a standardized, assay-independent, clinically validated troponin AUC method. The field should therefore refine biomarker-based approaches rather than assume that diagnostic troponin algorithms solve the infarct-sizing problem.

Natriuretic peptides and complementary biomarker risk stratification

Natriuretic peptides are relevant to this discussion because infarct-size estimation and post-MI risk stratification are related but not identical goals. B-type natriuretic peptide (BNP) and N-terminal pro-B-type natriuretic peptide (NT-proBNP) are not direct necrosis markers in the way CK-MB and troponin are; rather, they reflect ventricular wall stress, hemodynamic burden, and evolving ventricular dysfunction. For that reason, they are best viewed as complementary prognostic biomarkers rather than direct replacements for CK-MB AUC.

Several studies support their complementary role. Mayr et al. found that log-transformed NT-proBNP measured approximately day three after acute MI correlated with acute and chronic CMR infarct size as a percentage of left ventricular mass (r=0.59 to 0.64; p<0.004) and with ejection fraction (r=-0.49 to -0.55; p<0.004), while higher NT-proBNP was associated with lack of functional recovery [60]. Ceriani et al. reported that BNP and ANP were associated with infarct size measured by 99mTc-sestamibi gated SPECT, with BNP showing a significant association with perfusion-defect size in both univariate and multivariate models [61]. In a post-discharge acute myocardial infarction (AMI) cohort, short-term follow-up BNP predicted all-cause mortality (odds ratio: 2.265, 95% CI: 1.455-3.527) and major adverse cardiovascular events (odds ratio: 1.43, 95% CI: 1.101-1.858) after covariate adjustment [62].

These data suggest that an ideal biomarker strategy may require separation of two tasks: quantifying necrotic mass and predicting downstream remodeling or heart failure risk. CK-MB AUC is historically stronger for the first task, while natriuretic peptides may strengthen the second. A contemporary multi-marker model could therefore combine short-window necrosis markers, troponin kinetics, and natriuretic peptides to estimate both infarct burden and post-MI clinical risk.

Future directions

Future progress will likely require moving beyond the search for a single direct replacement biomarker. One approach is kinetic modeling of dense serial biomarker measurements. High-frequency sampling could allow investigators to model rise, peak, clearance, and curve morphology while adjusting for baseline troponin elevation, renal function, reperfusion status, and patient-specific factors. Kinetic modeling of myocardial necrosis biomarkers has already been proposed as a way to improve infarct-size estimation and evaluate conditioning therapies [7].

Machine-learning approaches may also help extract quantitative information from complex biomarker trajectories. Algorithms trained on serial troponin values, CK-MB, where available, natriuretic peptides, clinical variables, electrocardiographic features, angiographic data, and imaging endpoints may identify latent patterns that correlate with infarct size better than peak concentration or simple AUC calculations. Contemporary machine-learning models have improved diagnostic classification using troponin concentrations, but analogous models for infarct-size estimation require prospective validation against standardized imaging endpoints [63].

A second strategy is multi-marker assessment. Troponin could be combined with shorter half-life biomarkers, including CK-MB, heart-type fatty acid-binding protein, myoglobin, or other rapidly cleared markers, to capture different phases of myocardial injury. Early rule-in/rule-out troponin algorithms demonstrate the power of timed biomarker interpretation for diagnosis; similarly, timed strategies could be developed for infarct-size estimation rather than diagnosis alone [64].

Future validation studies should be designed specifically for quantification. Ideal studies would enroll well-phenotyped STEMI and NSTEMI cohorts; obtain serial biomarker sampling early enough to model rise, peak, and clearance; record renal function, baseline troponin elevation, reperfusion timing, and procedural variables; and compare biomarker-derived estimates against standardized CMR or SPECT infarct-size endpoints. NSTEMI cohorts should be deliberately powered rather than included as small secondary subgroups.

Proposed integrated framework

**Figure 1:**
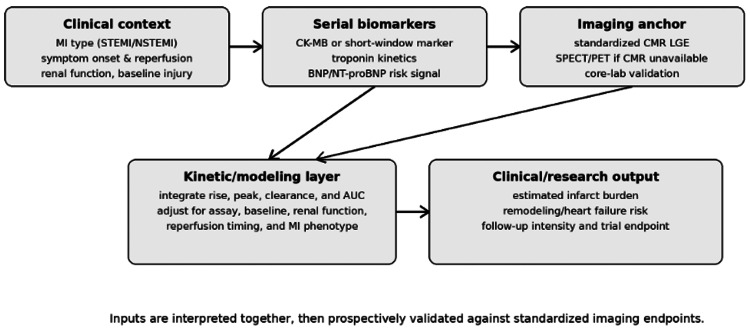
Proposed integrated biomarker-imaging framework for future infarct-size estimation BNP: B-type natriuretic peptide; CK-MB AUC: creatine kinase-myocardial band area under the curve; CMR: cardiac magnetic resonance; LGE: late gadolinium enhancement; STEMI: ST-segment elevation myocardial infarction; MI: myocardial infarction

A practical future framework should integrate clinical context, serial biomarkers, kinetic modeling, and imaging validation rather than treating any single measurement as definitive. At presentation, the model should record MI phenotype, symptom-onset certainty, reperfusion timing, baseline troponin status, renal function, and procedural variables. Serial biomarker sampling could then combine a short-window necrosis marker, such as CK-MB or a replacement candidate, troponin kinetics for sensitive injury detection, and BNP or NT-proBNP for remodeling and heart-failure risk. These inputs should be analyzed through a kinetic model and prospectively anchored to standardized CMR or SPECT endpoints. The final output would be both a quantitative infarct-burden estimate and a post-MI risk category useful for follow-up planning and clinical-trial endpoints (Figure 1).

If modeling and multi-marker strategies remain insufficient, novel biomarkers specifically optimized for infarct-size quantification will be needed. Cardiac-enriched microRNAs have biologic relevance in MI and may provide additional information about myocardial injury and repair [65]. Glycogen phosphorylase BB and other early injury markers have also been investigated as markers of acute MI [66]. These candidates remain experimental, but they illustrate the broader path forward: biomarkers designed and validated for quantification, not merely detection.

## Conclusions

Troponin has appropriately replaced CK-MB as the dominant diagnostic biomarker for MI, but it has not replaced CK-MB AUC as a validated biochemical method for infarct-size estimation. Troponin's prolonged elevation, baseline variability, multiphasic release, and assay heterogeneity limit its quantitative utility for this purpose. CK-MB AUC, despite lower diagnostic specificity, offered a shorter and more interpretable kinetic window that aligned more closely with myocardial necrosis. Imaging, particularly CMR with LGE, remains the contemporary reference standard for infarct-size quantification, but cost, access, timing, and workflow limitations restrict its use as a universal solution. NSTEMI further underscores the need for a dedicated biomarker strategy because smaller, subendocardial, and temporally heterogeneous infarcts are harder to size using simple biomarker assumptions derived from STEMI cohorts.

A scalable biomarker-based approach would improve risk stratification, standardize clinical-trial endpoints, and reduce dependence on advanced imaging where imaging is impractical or unavailable. The most promising path is not a return to CK-MB as a diagnostic competitor to troponin, but a modernized quantitative framework that integrates CK-MB or other short-window necrosis markers, troponin kinetics, natriuretic peptides for remodeling risk, computational modeling, and imaging-validated endpoints. Until such methods are established, contemporary cardiology remains without a practical, validated, bedside tool that fully replaces the infarct-sizing role once served by serial CK-MB AUC.
